# The Diversity of Yellow-Related Proteins in Sand Flies (Diptera: Psychodidae)

**DOI:** 10.1371/journal.pone.0166191

**Published:** 2016-11-03

**Authors:** Michal Sima, Marian Novotny, Lukas Pravda, Petra Sumova, Iva Rohousova, Petr Volf

**Affiliations:** 1 Department of Parasitology, Faculty of Science, Charles University, Prague, Czech Republic; 2 Department of Cell Biology, Faculty of Science, Charles University, Prague, Czech Republic; 3 CEITEC—Central European Institute of Technology, Masaryk University, Brno, Czech Republic; 4 National Centre for Biomolecular Research, Faculty of Science, Masaryk University, Brno, Czech Republic; Instituto Oswaldo Cruz, BRAZIL

## Abstract

Yellow-related proteins (YRPs) present in sand fly saliva act as affinity binders of bioamines, and help the fly to complete a bloodmeal by scavenging the physiological signals of damaged cells. They are also the main antigens in sand fly saliva and their recombinant form is used as a marker of host exposure to sand flies. Moreover, several salivary proteins and plasmids coding these proteins induce strong immune response in hosts bitten by sand flies and are being used to design protecting vaccines against *Leishmania* parasites. In this study, thirty two 3D models of different yellow-related proteins from thirteen sand fly species of two genera were constructed based on the known protein structure from *Lutzomyia longipalpis*. We also studied evolutionary relationships among species based on protein sequences as well as sequence and structural variability of their ligand-binding site. All of these 33 sand fly YRPs shared a similar structure, including a unique tunnel that connects the ligand-binding site with the solvent by two independent paths. However, intraspecific modifications found among these proteins affects the charges of the entrances to the tunnel, the length of the tunnel and its hydrophobicity. We suggest that these structural and sequential differences influence the ligand-binding abilities of these proteins and provide sand flies with a greater number of YRP paralogs with more nuanced answers to bioamines. All these characteristics allow us to better evaluate these proteins with respect to their potential use as part of anti-*Leishmania* vaccines or as an antigen to measure host exposure to sand flies.

## Introduction

During intake of a bloodmeal, sand flies (Diptera: Phlebotominae) and other bloodsucking insects inject saliva into the host skin. This saliva contains a mixture of various proteins, which play a major role in preventing host haemostatic and inflammatory responses of different pathways, e.g. platelet activation, coagulation, inflammation, mast cell function, and vasoconstriction (reviewed in [1]). In sand flies, the vectors of *Leishmania* protozoan parasites, these salivary proteins have been studied for decades due to their biological activities and possible use in anti-*Leishmania* vaccines (reviewed in [2]). In repeatedly bitten hosts, several salivary proteins elicit a strong antibody response, which can be utilized for the detection of exposure to sand flies in epidemiological studies [3–7].

Transcripts of yellow-related proteins (YRPs) have been present in the salivary cDNA libraries of all sand fly species tested to date [8–21]. Usually, they are found in more than one homolog, which may occur in N-glycosylated, O-glycosylated, C-glycosylated, or non-glycosylated forms [16, 20].

YRPs are known as (1) kratagonists that remove small molecule mediators of haemostasis by high affinity ligand-binding proteins, and as (2) antigens that elicit a host immune response, both antibody and cell-mediated. All sequences of sand fly YRPs contain the entire insect-specific MRJP (major royal jelly protein) domain, which defines this protein family across several insect orders/families including *Drosophila melanogaster* [22], honeybees [23], mosquitoes [24], and tse-tse flies [25]. In *Lutzomyia longipalpis*, Xu et al. [26] demonstrated that YRPs are high affinity binders of pro-haemostatic and pro-inflammatory biogenic amines, such as serotonin, histamine and catecholamines. Blocking of these small molecules by YRPs results in vasodilatation, platelet deactivation, and a decrease in vascular permeability [27, 28]. The YRP of *Phlebotomus duboscqi* was shown to have lectin-like activity and is swallowed into the midgut, together with saliva [29].

Sand fly salivary recombinant YRPs are the most promising antigens for measuring exposure in naturally bitten hosts, and have been the subject of large epidemiological studies [30–34]. Besides eliciting an antibody response, YRPs as well as plasmids coding these proteins of *Lutzomyia longipalpis* induce a strong delayed type hypersensitivity (DTH) reaction, which leads to protection against *Leishmania major* in vivo [26, 35] and against *L*. *infantum* in vitro [36]. This suggests a possible use of these proteins in an anti-*Leishmania* vaccine.

In 2011, the crystal structure of *L*. *longipalpis* YRP LJM11 (GenBank ACCN: AAS05318) was published as 3Q6K (Protein Data Bank ID) with a description of the ligand-binding pocket [26]. Based on this structure and available amino acid sequences obtained from GenBank, we constructed 3D models of all YRPs identified so far in sand fly sialomes. We predicted their phylogenetic relationships, glycosylation sites, surface electrostatic potentials, compared their sequences and characterized the ligand-binding tunnel. Our results show differences among individual proteins within one species as well as differences among various species. Our results may lead to a better understanding of the biological function of YRPs.

## Methods

### Phylogenetic analysis

Amino acid sequences of YRPs were identified in public databases at NCBI using BLAST [37] based on similarity with *Lutzomyia longipalpis* LJM11 (Protein Data Bank: 3Q6K, for the purpose of this study called 3Q6K_Llon1), the best explored protein from this group, [26]. All these analyses were performed for the sequences without a signal peptide, which was identified using SignalP 4.0 [38]. Sequences were consequently aligned using ClustalX (version 2.0) [39]. The best substitution matrix for creating a phylogenetic tree of sand fly salivary YRPs was determined in ProtTest software 2.0 [40]. TREEPUZZLE 5.2 [41] was used to create a maximum likelihood phylogenetic tree from the protein alignment using the WAG model [42] and quartet puzzling with 10000 puzzling steps. The resulting tree for all 31 proteins from 11 sand fly species in two genera was visualized in MEGA 4 [43] and rooted by the related protein from *Drosophila melanogaster* (ACCN: NP650247). Clustal Omega [44] was used with default settings to calculate a Percent Identity Matrix among all sand fly salivary YRPs.

### Prediction of glycosylation

Putative N-, O-, and C- glycosylation sites for all 31 protein sequences were determined using NetNGlyc 1.0, NetOGlyc 4.0 [45], and NetCGlyc 1.0 [46] servers with default settings.

### 3D models construction

All proteins were modeled using 3Q6K, the only available structure of sand fly salivary YRP in Protein Data Bank (PDB, [47]), from where its PDB file and fasta sequence were downloaded. The alignment of template and target sequence was done in Clustal Omega [44] for all proteins. Scripts for Python version 2.7 (Python Software Foundation) were prepared in a txt file and ran in MODELLER [48]. Five models were calculated for each protein, with the best one chosen based on the lowest energy levels of molpdf and the DOPE score. All models were displayed and analyzed in PyMOL (The PyMOL Molecular Graphics System, Version 1.5 Schrödinger, LLC.). Electrostatic surface potentials were calculated using the APBS Tools2 plugin [49] in PyMOL.

### Tunnel analysis

Tunnels in the protein structures were detected and characterized using the channel analysis tool MOLE 2.0 [50]. All proteins were superimposed using PyMOL in order to use the same settings, and therefore obtain comparable tunnels. In brief, MOLE 2.0 calculates a Delaunay triangulation/Voronoi diagram of the atomic centers. Next, tunnels are identified between every tuple of user defined end points within a protein structure using Dijkstra’s algorithm. The resulting tunnels are defined by their centerline and are uniformly divided into layers. Each layer is defined by the residues lining it. A new layer starts whenever there is a change in residues lining it along its length. Additionally, MOLE 2.0 inferrs basic physicochemical properties for each tunnel as well as layers. These were calculated from a unique set of lining residues averaging tabulated values for hydrophobicity [51]. WebLogo 2.8.2. [52] and Clustal Omega [44] were used to visualize differences among protein tunnels.

## Results

### Identification of sand fly salivary YRPs

Thirty two sand fly salivary YRPs were identified based on their similarity with PDB ID: 3Q6K from *L*. *longipalpis* [26], the only known 3D structure of YRPs of phlebotominae sand flies. These YRPs were detected in all 13 sand fly species with a published salivary gland transciptome: 9 *Phlebotomus* species from 5 subgenera (*Phlebotomus*, *Paraphlebotomus*, *Larroussius*, *Adlerius*, and *Euphlebotomus*), and 4 *Lutzomyia* species from 3 subgenera (*Lutzomyia*, *Nyssomyia*, and *Helcocyrtomyia*). In summary, there are currently 33 different YRPs described in the Phlebotominae subfamily (Table 1). There are large differences in the number of these proteins found in various species, ranging from 1 in *P*. *arabicus* and *P*. *argentipes* to 5 in *P*. *sergenti*.

**Table 1:** Identified sand fly salivary YRPs.

ACCN or name	Sand fly species	Subgenus	Published by	Identifier
BAM69109	*L*. *ayacuchensis*	*Helcocyrtomyia*	[19]	Laya1
BAM69110	*L*. *ayacuchensis*	*Helcocyrtomyia*	[19]	Laya2
BAM69111	*L*. *ayacuchensis*	*Helcocyrtomyia*	[19]	Laya3
BAM69185	*L*. *ayacuchensis*	*Helcocyrtomyia*	[19]	Laya4
AFP99235	*L*. *intermedia*	*Nyssomyia*	[17]	Lint
AAS05318, LJM11, 3Q6K	*L*. *longipalpis*	*Lutzomyia*	[10, 25]	3Q6K_Llon1
AAD32198, LJM17	*L*. *longipalpis*	*Lutzomyia*	[8]	Llon2
ABB00904, LJM111	*L*. *longipalpis*	*Lutzomyia*	[10]	Llon3
ANW11467	*L*. *olmeca*	*Nyssomyia*	[21]	Lolm1
ANW11468	*L*. *olmeca*	*Nyssomyia*	[21]	Lolm2
ANW11469	*L*. *olmeca*	*Nyssomyia*	[21]	Lolm3
ACS93501	*P*. *arabicus*	*Adlerius*	[14]	Para
ABA12136	*P*. *argentipes*	*Euphlebotomus*	[12]	Parg
AAX44093	*P*. *ariasi*	*Larroussius*	[11]	Pari1
AAX56360	*P*. *ariasi*	*Larroussius*	[11]	Pari2
ABI15938	*P*. *duboscqi*	*Phlebotomus*	[13]	Pdub1
ABI15941	*P*. *duboscqi*	*Phlebotomus*	[13]	Pdub2
ABI20172	*P*. *duboscqi*	*Phlebotomus*	[13]	Pdub3
AGT96460	*P*. *orientalis*	*Larroussius*	[20]	Pori1
AGT96461	*P*. *orientalis*	*Larroussius*	[20]	Pori2
AAL11051	*P*. *papatasi*	*Phlebotomus*	[9]	Ppap1
AAL11052	*P*. *papatasi*	*Phlebotomus*	[9]	Ppap2
AGE83094	*P*. *papatasi*	*Phlebotomus*	[15]	Ppap3
AGE83095	*P*. *papatasi*	*Phlebotomus*	[15]	Ppap4
ABA43049	*P*. *perniciosus*	*Larroussius*	[12]	Pper1
ABA43050	*P*. *perniciosus*	*Larroussius*	[12]	Pper2
ADJ54114	*P*. *sergenti*	*Paraphlebotomus*	[16]	Pser1
ADJ54115	*P*. *sergenti*	*Paraphlebotomus*	[16]	Pser2
ADJ54116	*P*. *sergenti*	*Paraphlebotomus*	[16]	Pser3
ADJ54122	*P*. *sergenti*	*Paraphlebotomus*	[16]	Pser4
ADJ54123	*P*. *sergenti*	*Paraphlebotomus*	[16]	Pser5
ADJ54079	*P*. *tobbi*	*Larroussius*	[16]	Ptob1
ADJ54080	*P*. *tobbi*	*Larroussius*	[16]	Ptob2

Sequence GenBank accession numbers (or names), sand fly species (ordered alphabetically), references, and identifier names used in this study are provided for each protein.

### Phylogenetic analysis

**Fig 1:**
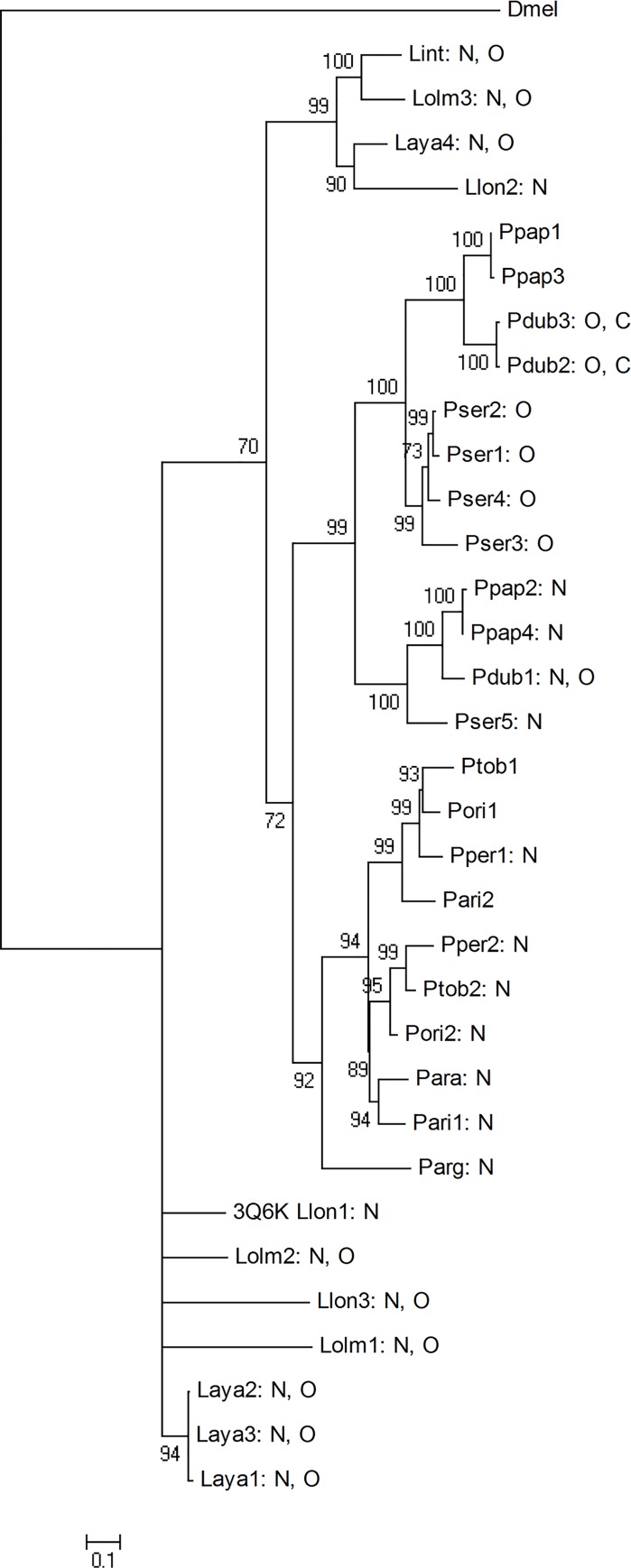
Phylogenetic tree of 33 sand fly salivary YRPs with putative glycosylation sites marked. The maximum likelihood phylogenetic tree was created in TREEPUZZLE with WAG model using quartet puzzling with 10000 puzzling steps. For rooting, the related protein (ACCN: NP650247) from *Drosophila melanogaster* (Dmel) was used. Bootstraps with support for branching are shown. The letters N, O, and C indicate putative N-, O-, and C-glycosylation sites, respectively. Protein codes refer to Table 1.

The phylogenetic analysis was performed using the maximum likelihood phylogenetic tree of all 33 YRPs from 13 sand fly species. A higher variability in *Lutzomyia* YRPs compared to *Phlebotomus* proteins was found: two clusters from seven *Lutzomyia* YRPs were detected but four other *Lutzomyia* proteins created own branches. Twenty two *Phlebotomus* proteins clustered together in one branch with high bootstrap support. A closer phylogenetic relationship was discovered between the subgenera *Phlebotomus* (*P*. *papatasi* and *P*. *duboscqi*) and *Paraphlebotomus* (*P*. *sergenti*), as well as between *Larrousius* (*P*. *tobbi*, *P*. *orientalis*, *P*. *perniciosus*, and *P*. *ariasi*) and *Adlerius* (*P*. *arabicus*) species. *Euphlebotomus* (*P*. *argentipes*) protein created a single branch, which is closer to species from subgenera *Larroussius* and *Adlerius* (Fig 1).

Based on the identity matrix from Clustal Omega, the similarity among all 33 YRPs (528 combinations) varied from 44 to 99% (S1 Table).

### Glycosylation

For all 33 proteins, potential N-, O- and C-glycosylation sites were predicted. In 11 proteins, only N-glycosylation sites were predicted. In four YRPs of *P*. *sergenti* (Pser1-Pser4), only O-glycosylation sites were identified, and C-glycosylation sites were predicted for only two proteins from *P*. *duboscqi* (Table 2). In eleven proteins, more than one type of putative glycosylation was found.

**Table 2:** Putative glycosylation sites.

Identifier	N-glycosylation	O-glycosylation	C-glycosylation
Laya1	Asn 164, Asn 196	Ser 208	-
Laya2	Asn 164, Asn 196	Ser 113, Ser 208	-
Laya3	Asn 164, Asn 196	Ser 208	-
Laya4	Asn 11, Asn 264	Ser 262	-
Lint	Asn 11	Thr 262, Thr 263	-
3Q6K_Llon1	Asn 195	-	-
Llon2	Asn 11	-	-
Llon3	Asn 123	Ser 300	-
Lolm1	Asn 122	Thr 262	-
Lolm2	Asn 194	Thr 261	-
Lolm3	Asn 11	Thr 197	-
Para	Asn 11	-	-
Parg	Asn 11, Asn 18, Asn 307	-	-
Pari1	Asn 11, Asn 201	-	-
Pari2	-	-	-
Pdub1	Asn 11, Asn 65	Thr 367	-
Pdub2	-	Ser 208	Trp 338
Pdub3	-	Ser 208	Trp 338
Pori1	-	-	-
Pori2	Asn 11	-	-
Ppap1	-	-	-
Ppap2	Asn 11, Asn 65	-	-
Ppap3	-	-	-
Ppap4	Asn 11, Asn 65	Ser 264	-
Pper1	Asn 11	-	-
Pper2	Asn 11	-	-
Pser1	-	Ser 81, Ser 208, Thr 263	-
Pser2	-	Ser 208, Thr 263	-
Pser3	-	Ser 208, Thr 263	-
Pser4	-	Ser 208	-
Pser5	Asn 11, Asn 65, Asn 250	-	-
Ptob1	-	-	-
Ptob2	Asn 11	-	-

N-, O- and C-glycosylation sites were predicted for each protein using glycosylation servers. Numbers and three-letter abbreviations indicate the positions of predicted glycosylation and the amino acid where the glycosylation occurs (Asn–asparagine, Thr–threonine, Ser–serine, and Trp–tryptophan).—shows cases where no glycosylation sites were identified. Protein codes refer to Table 1.

The predicted glycosylation sites for the majority of YRPs (77% and 53% of putative N-glycans and O-glycans, respectively) share the same positions (Table 2). Importantly, proteins clustering together in the phylogenetic tree (Fig 1) had similar predicted glycosylation patterns.

### 3D model analysis

**Fig 2:**
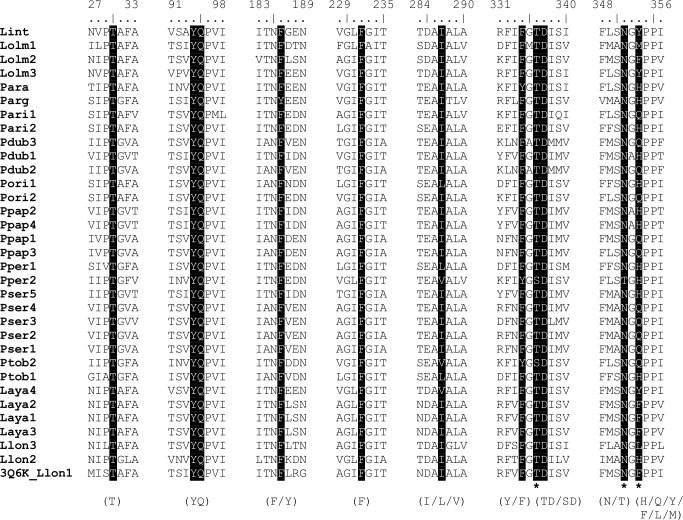
Amino acid alignment within the YRP ligand-binding sites. The alignment was created in ClustalX, and shows high conservancy in binding sites (letters in brackets below the sequences, slashes indicate amino acid substitutions in appropriate positions). Ligand binding sites are highlighted in black, three bold stars below the sequences represent the main binding sites where hydrogen bonds between amino acids and the ligand are predicted. Protein codes refer to Table 1.

The amino acid sequence alignment of 33 sand fly salivary YRPs showed high conservancy in several regions, with 57 invariant sites identified among all sequences, representing approximately 15% of the total sequence. Five out of eleven amino acids creating the ligand-binding pocket known from Xu et al. [26] were identical across all proteins. The most variable position in the ligand-binding site was the one annotated as Phe 344 in the crystal structure of YRP 3Q6K—this position is occupied by six different amino acids (His, Gln, Tyr, Phe, Met, or Lys) in our studied set of proteins. Two most common amino acids on this position are histidine and glutamine (Fig 2).

**Fig 3:**
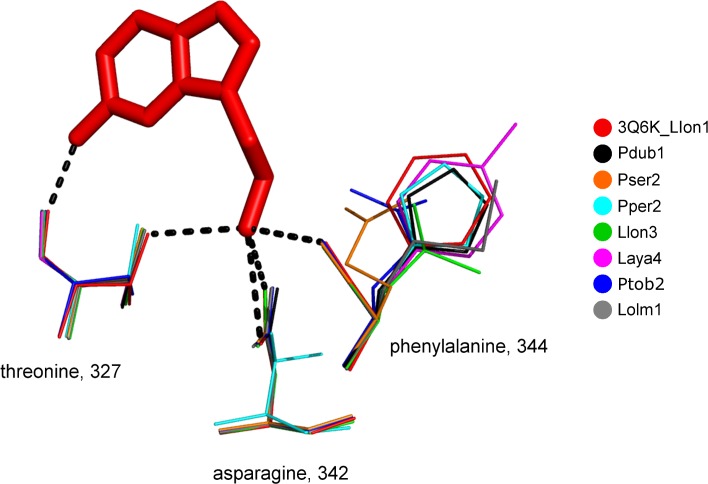
Hydrogen-binding between serotonin and ligand-binding amino acids in sand fly salivary YRPs. The figure shows the variability in hydrogen bonds between serotonin and 3Q6K (provided by Thr 327, Asn 342, and Phe 344) described in Xu et al. [26] and other YRP models created during this study and visualized in PyMOL. The red bold molecule symbolizes the ligand serotonin and the black dashed line show the hydrogen bonds between amino acids and serotonin in 3Q6K. Other colors indicate one representative member of one protein group. A group was defined as having at least one unique mutation from the template structure in one of the three described amino acids binding the ligand. From each group, the protein with the most abundant amino acid rotamer was chosen as being representative for this visualization. Protein codes refer to Table 1.

Models for 32 different proteins were created based on the template sequence 3Q6K from *L*. *longipalpis* (S1 Fig). All of them folded as a six-bladed beta-propeller, similarly as in 3Q6K [26]. In 3Q6K, 11 ligand-binding amino acids were identified [26]. Three of them (in the case of 3Q6K –Thr 327 with 2 bonds, Asn 342 with 2 bonds, and Phe 344 with 1 bond) play a major role in binding abilities, because of the hydrogen bonds between the protein amino acids and the ligand. The other 8 amino acids bind serotonin by Van der Waals or hydrophobic interactions. These three amino acids were visualized in the 3D alignment of all 33 proteins. Nine different amino acids were found at three positions that create hydrogen bonds with serotonin from 3Q6K (Fig 3).

As well as in 3Q6K_Llon1, in Laya1-3 and Lolm1, the amino acids creating hydrogen bonds with serotonin are Thr 327, Asn 342, and Phe 344 (using the numbering for 3Q6K). The largest group includes 11 proteins (Llon2, Para, Parg, Pari, Pdub1, Pori2, Ppap2, Ppap4, Pper1, Pser5, and Ptob1). In comparison with the group containing 3Q6K_Llon1, there is a substitution of histidine instead of phenylalanine in the last binding position. A substitution in the same position (glutamine instead of phenylalanine) also occurs in the second biggest group, which includes 10 proteins (Pari1, Pdub2, Pdub3, Pori2, Ppap1, Ppap3, Pser1, Pser2, Pser3, and Pser4). The last group with more than one protein (Laya4, Lint, and Lolm3) has substitution in the last binding position, as well. In this case, there is tyrosine instead of phenylalanine. Pper2 has substitutions in all three positions (serine, threonine, and histidine). Because of these substitutions (mainly due to the presence of Thr instead of Asn) these proteins are likely to have different binding ability to biamines from 3Q6K_Llon1. In the last binding positions of Llon3 and Lolm1 there are leucine and methionine, respectively, instead of phenylalanine. Ptob2 contains a substitution in the first binding position, with threonine replaced by serine. The distribution of YRPs in these groups partially corresponds with branches in the phylogenetic tree (S2 Fig).

### Tunnel in YRPs

In Xu et al. [26], YRP 3Q6K from *L*. *longipalpis* was described as a hollow barrel with two possible entrances. Here, based on the models created using other sand fly YRPs and MOLE analysis, we found a very similar hollow barrel in all proteins we studied (Table 3). It is therefore likely that all studied proteins retain the ability to bind a ligand. The differences in a length and minimal radius of the identified tunnels have limited relevance due to the fact that they are calculated on theoretical models. On contrary, differences in hydrophobicity should be less influenced by homology modeling process and could therefore indicate (where present) changes in binding affinities among paralogs within species.

**Table 3:** A comparison of tunnels in sand fly salivary YRPs.

Identificator	Length	Min. Radius	Hydrophobicity	Ligand-side	Opposite-side
Laya1	38.320	1.769	-0.13	Positive	Negative
Laya2	38.707	1.813	-0.22	Positive	Negative
Laya3	41.045	0.962	-0.17	Positive	Negative
Laya4	41.295	1.785	-0.25	Neutral (negative)	Neutral (negative)
Lint	39.194	1.539	-0.32	Negative	Negative
3Q6K_Llon1	41.245	1.892	-0.23	Positive	Neutral (negative)
Llon2	39.722	1.361	-0.07	Negative	Negative
Llon3	43.634	1.831	-0.29	Negative	Negative
Lolm1	38.595	2.055	-0.21	Negative (neutral)	Neutral
Lolm2	41.696	1.997	-0.45	Positive (neutral)	Neutral (negative)
Lolm3	43.098	1.416	-0.86	Neutral (negative)	Negative (neutral)
Para	45.294	1.229	-0.53	Negative	Negative
Parg	40.506	1.262	-0.20	Negative	Negative
Pari1	45.068	1.673	-0.02	Negative	Negative
Pari2	41.717	1.880	-0.29	Negative	Negative
Pdub1	37.292	1.744	-0.14	Negative	Neutral (negative)
Pdub2	39.912	1.433	0.03	Neutral (positive)	Neutral (negative)
Pdub3	35.967	2.021	-0.34	Negative	Neutral (positive, negative)
Pori1	42.203	1.677	-0.22	Negative	Negative
Pori2	32.368	1.824	-0.18	Negative	Neutral (negative)
Ppap1	38.163	1.834	-0.23	Negative	Neutral (positive)
Ppap2	40.508	1.780	-0.05	Neutral (negative)	Negative (neutral)
Ppap3	40.849	2.053	-0.08	Negative	Neutral (positive)
Ppap4	46.137	1.518	-0.27	Neutral	Neutral (negative, positive)
Pper1	41.652	1.419	-0.18	Negative	Negative
Pper2	41.944	1.948	-0.25	Neutral (negative)	Neutral (negative)
Pser1	50.023	2.047	-0.27	Negative	Neutral
Pser2	37.843	1.755	-0.05	Negative	Neutral
Pser3	38.013	1.757	0.02	Neutral	Negative
Pser4	31.949	1.812	0.01	Negative	Negative
Pser5	40.761	1.853	-0.01	Negative	Neutral
Ptob1	33.603	1.740	-0.31	Negative	Negative
Ptob2	34.118	1.822	-0.22	Neutral (positive)	Neutral (negative)

The length and minimum radius (in Ångströms) and hydrophobicity are shown for the tunnels of each protein. All values were calculated using MOLE. Surface electrostatic potentials around protein entry sides were calculated in PyMOL using the APBS tool. "Ligand-side" refers to the side with the shorter distance from the beginning of the tunnel to the ligand-binding site, while "Opposite-side" is the other side of the tunnel. The most common electrostatic surface potentials of both entrances to the tunnel are shown. Charges in brackets indicate that the charge around the entrance is not uniform, and small parts of the entrance are charged differently from the majority of the entrance. Protein codes refer to Table 1.

**Fig 4:**
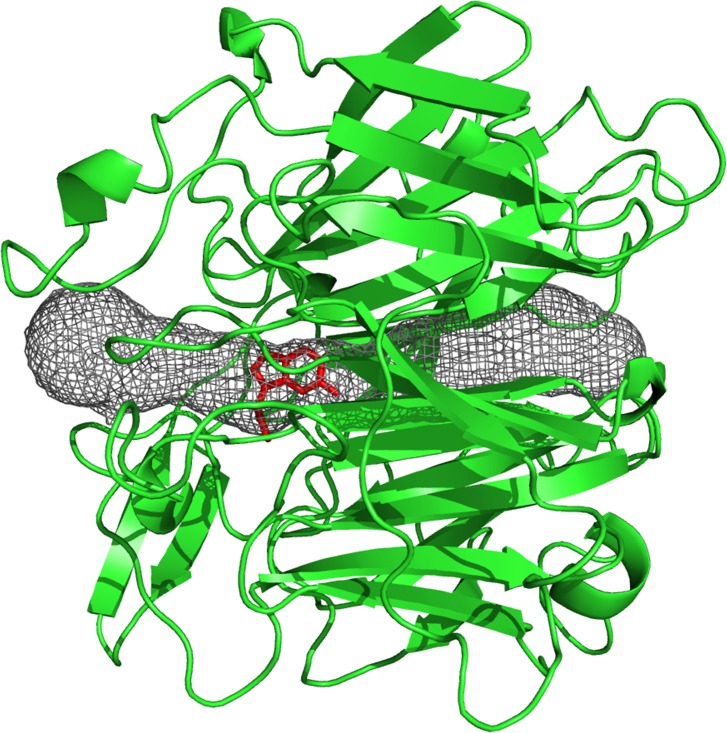
Visualization of the tunnel in 3Q6K_Llon1. The structure was visualized in PyMOL using the MOLE script for calculating the tunnel. The 3Q6K_Llon1 protein is drawn in green, the tunnel through this protein structure is in black mesh and the red-stick molecule represents serotonin.

The ligand-binding site for serotonin in 3Q6K is located approximately one third of the way along this tunnel (Fig 4). The comparison based on the APBS tools in PyMol of all models and the 3Q6K structure revealed that the surface electrostatic potential of the entrance closer to the binding site varies from negative to positive (S2 Fig), and the size of this entrance is smaller than the opposite entrance. In contrast, the entrance more distant from the binding site is larger and neutrally or negatively charged in all these proteins (Table 3). The distribution of the surface potentials in most of these proteins corresponds with the phylogenetic relationships (Fig 1) and predicted glycosylation sites (Table 2).

**Fig 5:**
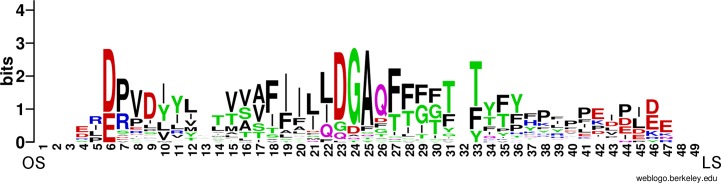
Sequence conservation of the amino acids lining the ligand-binding tunnel. The WebLogo server was used to visualize the sequence conservation of the amino acids lining the ligand-binding tunnel in 31 YRPs. The amino acids are colored according to their physical-chemical properties. LS and OS below the x-axis indicate the side closer to the ligand-binding site and the opposite side of the tunnel, respectively.

The sequence conservation of the amino acids lining the tunnels was explored using WebLogo server (Fig 5). The WebLogo was based on the multiple sequence alignment (S3 Fig) of all amino acids lining the accessible tunnels towards the ligand-binding site, as described by MOLE. The analysis showed that there are not any absolutely conserved amino acids, but both entrances to the tunnel are dominated by negatively-charged residues; however, there are proteins (e.g. Pdub1, Pari2, Ppar1, Ppap4, and Pser5) that are positively charged at least at one of the entrances. The middle section of the tunnel lining is occupied mostly by hydrophobic and small polar amino acids.

## Discussion

Here we focused on the bioinformatic characterization of sand fly salivary YRPs. These proteins have high affinity binding properties for pro-haemostatic and pro-inflammatory biogenic amines [26], but they are also highly antigenic for hosts bitten by sand flies [30–34] and can be used for anti-*Leishmania* vaccine development [53]. We searched published transcriptomes and recombinant proteins and found 33 different proteins from this family among 13 species from the two genera–Old World *Phlebotomus* and New World *Lutzomyia*. We analyzed their phylogenetic relationships, predicted glycosylation sites, constructed 3D models, identified tunnels passing through these structures, described the ligand-binding site, and compared their surface electrostatic potentials. The number of detected YRPs in these 13 sand fly species varied from one in *P*. *arabicus* [14], *P*. *argentipes* [12], and *L*. *intermedia* [17] to five in *P*. *sergenti* [16]. Such interspecies variability might be partially attributed to the sensitivity of sequencing methods; however, this is not likely the case of YRPs that were analyzed in the same laboratory using the same method. For example, Anderson et al. [12] reported a single YRP in *P*. *argentipes* but two in *P*. *perniciosus*, while Rohousova et al. [16] reported two in *P*. *tobbi* but five in *P*. *sergenti*. It is also possible that occurence of various numbers of YRPs is caused by differential gene expression. Genes with lower expression might be recorded less frequently as it was shown for other sand fly protein families [54].

Our phylogenetic analysis suggested a higher variability in *Lutzomyia* than in *Phlebotomus* YRPs. Sequence identity among YRPs ranged between 44 and 99 percent, with a majority of proteins having sequence identity around 50 percent. Further analysis divided these proteins into several branches, with closer relationships within sand flies from the same subgenera (*Phlebotomus*, *Paraphlebotomus*, and *Larrousius*). This confirmed previous analyses based on amino acid sequences [16, 20] and also corresponds with an analysis based on small subunit nuclear ribosomal DNA [55] showing two main clades–*Phlebotomus* with *Paraphlebotomus* subgenera clustering together and leaving other subgenera (e.g. *Euphlebotomus*, *Larroussius*, *and Adlerius*) in the second clade.

The biogenic function of proteins may be affected by the presence or absence of posttranslational modifications [56, 57]. For example, the antibody response against some glycoproteins can be directed strictly to the glycan part [58]. In accordance with our previous studies [16, 20, 54, 59], we showed both inter- as well as intra-species variability in the glycosylation of sand fly YRPs, which corresponded well with the phylogenetic analysis. Moreover, the same positions of putative glycosylation sites among YRPs mirrored the presumed effects of glycosylation on protein folding and stability, suggesting a highly conserved tertiary structure. It would be interesting to evaluate similarities in the antigenic potential of the proteins belonging to the same clusters.

The only protein with an experimentally solved 3D structure, LJM11 (3Q6K) from *Lutzomyia longipalpis*, has a unique six-bladed beta propeller fold that has only been identified in this protein. It is characterized by a long ligand-binding tunnel with two entrances–one on each side of the protein. Our 3D models of other YRPs showed similar structures as 3Q6K, and the tunnel is present in all studied proteins. Its length or minimum width vary between and within species but it remains uninterrupted in all modeled proteins, suggesting it is very important feature of these proteins. LJM11 is known to bind positively charged biogenic amines [26], and our structural analysis of YRPs from other sand fly species showed that in all tested sequences the ligand-binding tunnels were negatively charged and could therefore bind positively charged ligands of bioamine size. Nevertheless, we observed that the proteins and more specifically the paralogs within one species differ in the charge of tunnel entrances. Our data indicate that paralogs within one species will bind similar ligands with different affinities, and the ligands may travel different paths to the binding site according to the charge of the entrances. YRPs also differed within and among species in the length of their tunnels, the minimum radius of the tunnel and its hydrophobicity. We expect that all these parameters affect the nature and the affinity of the preferred ligand, which results in a greater binding versatility of the host bioamines.

In the 3Q6K YRP structure, the serotonin ligand is stabilized by hydrogen bonds from three amino acids in the main chain and side chain and by a number of additional hydrophobic interactions [26]. Both our sequence and structure analyses showed the diversity of this crucial site among the 33 proteins studied here. The largest diversity was observed on the position denoted in 3Q6K as Phe 344 (see Fig 3). The amino acid at this position participates in ligand-binding through a hydrogen bond from the main chain. Substitutions at this position should not play a major role in ligand-binding, yet it is the only amino acid of the ligand-binding-site that is different among three *L*. *longipalis* yellow-related proteins that have been shown to have different affinities for serotonin and other tested bioamines [26]. Our models of the other YRPs suggest that at least in the case of the Phe344Gln mutation, the glutamine side chain could provide a further hydrogen bond to the ligand (serotonin). Interestingly, our studied YRPs can be divided into several groups according to their ligand-binding composition, and some groups correlate with the branches identified in phylogenetic distribution as well as with the predicted glycosylation patterns. For example, Ppap2, Ppap4, Pdub1, and Pser5 (proteins from the largest group with binding amino acids Thr-Asn-His) or Pser1-4 (proteins from the second largest group with binding amino acids Thr-Asn-Glu) are located in two branches in the phylogenetic tree and share the same N-glycosylation and O-glycosylation pattern, respectively (see S2 Fig).

The lining of the tunnel is not conserved, but the entrances to the tunnel are composed mostly of negatively charged residues and the inner part of the tunnel is lined by hydrophobic and small polar amino acids (threonines). There are however several proteins (Pdub1, Ppap4, Pser5 etc.) that have positively charged residues forming one of the entrances (typically the one closer to ligand-binding site within the tunnel)–this might allow interaction with different ligands.

In summary, we found interesting diversity among these important proteins. All 33 sand fly salivary YRPs studied here share similar folding and contain a ligand-binding tunnel. Nevertheless, modifications found among these proteins influence the charge of entrances to the tunnel, the length of the tunnel and/or its hydrophobicity. We suggest that these modifications provide sand fly species with multiple paralogs of YRPs with more nuanced answers to host bioamines. Further experiments are needed to validate these models, but we assume, that our results should allow a better understanding of the biological role of these important proteins with respect to their potential use in anti-*Leishmania* vaccines or as host exposure markers to sand flies.

## Supporting Information

S1 Fig Modeled 3D structures of sand fly yellow-related proteins. Zipped PDB files of 32 sand fly yellow-related proteins calculated in MODELLER based on 3Q6K as the template structure. Protein codes refers to Table 1.
(ZIP)

S2 Fig Visual summary of the diversity of yellow-related proteins in sand flies. The maximum likelihood phylogenetic tree was created in TREEPUZZLE with WAG model using quartet puzzling with 10000 puzzling steps. For rooting, the related protein (ACCN: NP650247) from *Drosophila melanogaster* (Dmel) was used. Bootstraps with support for branching are shown. Protein codes refer to Table 1. Symbols preceding the protein codes indicate the most common surface electrostatic potential of the entrance to the protein tunnel closer to the ligand-binding site (LS) as shown in Table 3 (◊ positive, ○ neutral, and □ negative). Colors in symbols indicate the affiliation to protein groups with the same ligand-binding amino acids as shown in Fig 3. The letters N, O, and C indicate putative N-, O-, and C-glycosylation sites, respectively, based on Table 2.
(PDF)

S3 Fig Alignment of amino acids creating tunnels in yellow-related proteins. For each protein, amino acids creating tunnel were determined using MOLE. Clustal Omega was used to visualize differences of these protein tunnels. In sequences, small letters indicate main chain interaction and capital letters represent side chain interactions. Above the sequences, LS and OS describe the side closer to the ligand binding site and the opposite side of the tunnel, respectively. Protein codes refer to Table 1.
(PDF)

S1 Table Percent Identity Matrix of sand fly yellow-related proteins. Clustal Omega was used to calculate this sequence identity matrix among all identified yellow-related proteins. The similarity between the proteins is shown in percents. Different colors represent 10 percent similarity intervals. Protein codes refer to Table 1.
(PDF)
