# Supplementary figures and images for: The Diversity of Yellow-Related Proteins in Sand Flies (Diptera: Psychodidae)
Source: PLoS One. 2016 Nov 3;11(11):e0166191. doi: 10.1371/journal.pone.0166191 (PMC5094789; doi:10.1371/journal.pone.0166191)

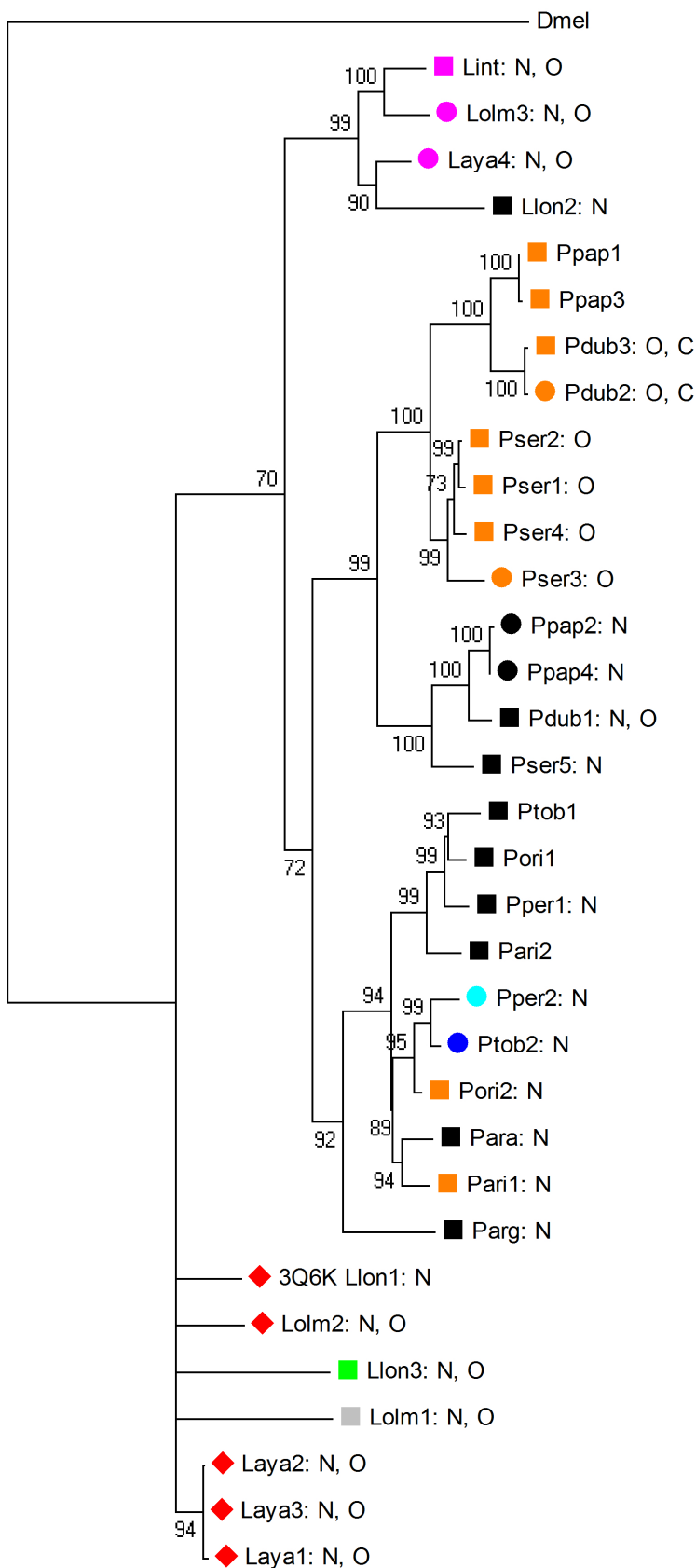

0.1

Supplement: S2 Fig — The maximum likelihood phylogenetic tree was created in TREEPUZZLE with WAG model using quartet puzzling with 10000 puzzling steps. For rooting, the related protein (ACCN: NP650247) from Drosophila melanogaster (Dmel) was used. Bootstraps with support for branching are shown. Protein codes refer to Table 1. Symbols preceding the protein codes indicate the most common surface electrostatic potential of the entrance to the protein tunnel closer to the ligand-binding site (LS) as shown in Table 3 (◊ positive, ○ neutral, and □ negative). Colors in symbols indicate the affiliation to protein groups with the same ligand-binding amino acids as shown in Fig 3. The letters N, O, and C indicate putative N-, O-, and C-glycosylation sites, respectively, based on Table 2. (PDF) [file pone.0166191.s002.pdf]
