# Supplementary material for: The Diversity of Yellow-Related Proteins in Sand Flies (Diptera: Psychodidae)
Source: PLoS One. 2016 Nov 3;11(11):e0166191. doi: 10.1371/journal.pone.0166191 (PMC5094789; doi:10.1371/journal.pone.0166191)
Supplement: S3 Fig — For each protein, amino acids creating tunnel were determined using MOLE. Clustal Omega was used to visualize differences of these protein tunnels. In sequences, small letters indicate main chain interaction and capital letters represent side chain interactions. Above the sequences, LS and OS describe the side closer to the ligand binding site and the opposite side of the tunnel, respectively. Protein codes refer to Table 1. (PDF) [file pone.0166191.s003.pdf]

```
                  OS                                                 LS
Lolm3             -----EAidyalRiTVSfii-lQgADfigFT-fYFPYRTEKE-------
Pper1             ------dEmiPRylTVSfii-lQgADflFgy-YTFPHLiEeE-------
Pser3             -----DPvdVyiMlVAtifTQllgAgfitIgFyYTFPKLipK-------
Llon2             -----DPVdyRLilvVAfaiDQggyYFfF--------vRVEEveEK---
Parg              ----iERPevylTAfVVaSCiQDTtfgcFtT-yYfYhHRpP-vDLd---
Llon3             ---EPdseiiRylTVVSfiliQDgfgitFTt-TlKYP-LFFElpEY---
Pdub2             -----DPVdyil--MVAfaigQDAftIFgty-T--YTPFKf--------
Lolm1             -----ESVdiylVTvTVfiSilDgAQftgFt-TTIYmKPFFEipd---
Laya2             -----DPVdiyT--VSvfiilQDgAftFgIT-tTfPKFYvpk-------
Lolm2             -----DPVdiyT--VSvV-fiQDgAftFgtT-YfFFKPvYpdD------
Pper2             -----EPvdyli--TVVSfi-QDgAsfgFSt-TYfFHPREPiIN-----
Laya3             -----DPVdiylTVvSAifi-lDQggFfYFP-FKvYk------------
Laya1             -----DPvdyil--VSTifi-lDgAQftIgF-fYFPFKYvpK-------
Para              -----EPdsiyilSTTVfii-lQgADfitFt-TITYhHRpPpvE-----
Pori1             -----EPsdvVi-yVTSfii-lDgAQflLFt-tTTFhPYHFELpieE--
3Q6K_Llon1        -----DPvdyli--TVSfii-lDgAQfitFt-TITfFYFPKYp-irR--
Ppap3             -----DPVdiylVMvAAfii-lDgAQtfIgT-FyFPqYIpEKiDLD---
Laya4             -----DPVKiyl-TVSvfii-lDgAQfvg-V-tFtTTYfPYFRpiEK--
Ptob1             -----EPVdyil-TVSvfii-lDgAQtfg-F-tyTYfFl----------
Ppap2             -----DPVd-iy-TVMvfii-lDgAQfFg-T-fYFlRDLPteIE-----
Ptob2             -----EPVd-iy-TVSvfii-lDgAQsfg-F-TfYFiRI----------
Lint              ---El-RySIiV--iStTiyilDgAQftIFg-T--fYFYPRp--iEnN-
Pori2             --------ilyV--SVAifaliDgAQfFgYf-F--RQPLLipdD-----
Pser5             -----DRRPlLv--MAaifaliDgAQftgFT-f--YFPHLiDLIdD---
Pari1             EPKRldelyiMV--tTiTfQligDAQftFgT-f--YFiIPLED-LKen-
Ppap4             ---DR-RPlMyT--LA-itfligDAQftIgF-t--ThPHRpptPleEdL
Pari2             ---ER-RPlyiV--TVTSifliDgAQftFtT-T--hHPYFLLEipeEE-
Pdub1             ---DR-RPlLvM--TAitfaligDQAftFtT-T--hYPHFL--------
Pdub3             ----idRPvVyl--MAA-limfDgAQtfFtT-TqPQYKfDp--------
Ppap1             -----DRRPlyM--LAA-liifDgAQtfFTI-FqPY-QIppEi--d---
Pser2             --------DlRy--MVA-ilfiDgAtQf-gF-T-yYFPQiLVd--D---
Pser1             DKEsPdvViyVM--AlA-ifliDgAQtfFgy-Y--yFPQKiDp------
Pser4             --------DvLM--Ala-ifliDgAQtfIgF-TtTYyPFQIi-------
```
