# Supplementary material for: The Diversity of Yellow-Related Proteins in Sand Flies (Diptera: Psychodidae)
Source: PLoS One. 2016 Nov 3;11(11):e0166191. doi: 10.1371/journal.pone.0166191 (PMC5094789; doi:10.1371/journal.pone.0166191)
Supplement: S1 Table — Clustal Omega was used to calculate this sequence identity matrix among all identified yellow-related proteins. The similarity between the proteins is shown in percents. Different colors represent 10 percent similarity intervals. Protein codes refer to Table 1. (PDF) [file pone.0166191.s004.pdf]

	Parg	Pari2	Ptob1	Pori1	Pper1	Pori2	Pper2	Ptob2	Para	Pari1	Pser5	Pdub1	Ppap2	Ppap4	Pser3	Pser4	Pser2	Pser1	Ppap1	Ppap3	Pdub3	Pdub2	Llon2	Laya4	Lint	Lolm3	Llon3	Lolm1	3Q6K_Llon1	Lolm2	Laya1	Laya2	Laya3
Parg	100.00	57.84	57.84	58.81	58.11	63.27	62.57	60.96	64.97	63.64	54.93	55.47	56.38	56.91	54.45	53.64	55.53	54.72	51.89	51.35	51.89	52.16	48.28	55.97	54.11	53.85	48.66	47.98	53.62	50.27	53.60	54.13	54.13
Pari2	57.84	100.00	80.54	84.01	82.43	78.38	70.54	74.59	78.11	77.84	53.26	51.89	52.16	52.70	56.68	56.95	56.40	56.40	53.55	53.55	52.46	52.46	45.80	53.66	53.12	53.39	49.59	50.82	52.45	49.86	51.22	52.03	52.03
Ptob1	57.84	80.54	100.00	87.26	87.30	75.68	70.00	72.16	72.70	72.16	51.90	50.27	50.27	50.81	55.04	54.50	54.22	54.22	50.55	50.55	50.55	50.55	44.17	51.49	51.76	50.95	51.23	48.91	51.36	49.05	50.68	51.49	51.49
Pori1	58.81	84.01	87.26	100.00	88.35	77.78	71.00	74.53	74.80	75.61	53.13	50.95	50.95	51.76	55.86	56.13	55.86	55.59	52.19	52.19	51.64	51.91	45.38	53.26	52.45	52.45	51.09	49.73	52.86	50.55	51.63	52.45	52.45
Pper1	58.11	82.43	87.30	88.35	100.00	77.03	70.81	73.24	75.14	76.76	54.35	51.62	51.35	52.16	56.68	56.40	56.13	56.13	52.46	52.46	53.01	53.01	46.34	55.01	53.39	52.85	51.50	50.00	51.09	49.32	50.68	51.49	51.49
Pori2	63.27	78.38	75.68	77.78	77.03	100.00	86.86	91.69	83.65	84.45	57.41	54.72	56.18	56.18	57.22	56.68	57.77	57.77	55.19	55.46	55.74	55.19	49.46	59.14	58.60	59.68	50.68	52.73	54.62	53.13	53.78	54.32	54.32
Pper2	62.57	70.54	70.00	71.00	70.81	86.86	100.00	90.13	81.55	80.21	56.72	53.76	55.23	55.23	54.35	53.26	54.35	54.35	51.23	51.50	52.04	51.77	49.33	56.57	54.69	56.03	50.00	50.95	51.76	51.63	52.56	53.10	53.10
Ptob2	60.96	74.59	72.16	74.53	73.24	91.69	90.13	100.00	81.82	81.55	55.65	54.84	55.50	55.50	54.89	54.35	55.43	55.43	53.95	54.22	53.13	52.86	47.45	56.84	54.42	56.84	49.18	51.23	52.30	51.36	52.02	52.56	52.56
Para	64.97	78.11	72.70	74.80	75.14	83.65	81.55	81.82	100.00	85.07	56.03	53.49	53.35	53.89	55.43	55.98	57.07	57.07	54.50	54.77	54.22	54.22	48.93	56.95	56.68	58.29	50.00	50.14	52.85	52.17	53.10	53.64	53.64
Pari1	63.64	77.84	72.16	75.61	76.76	84.45	80.21	81.55	85.07	100.00	56.30	54.30	54.96	55.50	57.34	55.98	57.07	57.07	54.50	54.77	54.22	53.95	48.40	56.42	56.15	55.61	50.27	52.59	52.30	51.36	52.02	52.56	52.56
Pser5	54.93	53.26	51.90	53.13	54.35	57.41	56.72	55.65	56.03	56.30	100.00	76.78	77.11	77.63	62.67	64.53	66.40	66.67	60.16	59.36	59.63	59.89	49.47	53.17	50.79	51.85	46.92	51.48	49.87	51.47	52.53	53.60	53.60
Pdub1	55.47	51.89	50.27	50.95	51.62	54.72	53.76	54.84	53.49	54.30	76.78	100.00	87.66	88.45	59.95	61.01	62.60	62.86	57.45	57.18	57.18	57.98	48.81	51.99	49.60	49.87	45.19	51.21	49.60	51.87	54.11	55.17	55.17
Ppap2	56.38	52.16	50.27	50.95	51.35	56.18	55.23	55.50	53.35	54.96	77.11	87.66	100.00	98.95	59.95	60.21	61.54	61.80	57.18	56.65	56.12	56.65	48.94	53.17	52.12	51.32	45.72	50.67	49.60	51.34	53.32	54.38	54.38
Ppap4	56.91	52.70	50.81	51.76	52.16	56.18	55.23	55.50	53.89	55.50	77.63	88.45	98.95	100.00	60.48	60.74	62.07	62.33	57.71	57.18	56.65	57.18	48.94	53.44	52.12	51.32	46.26	51.21	50.13	51.34	53.85	54.91	54.91
Pser3	54.45	56.68	55.04	55.86	56.68	57.22	54.35	54.89	55.43	57.34	62.67	59.95	59.95	60.48	100.00	89.66	87.27	87.00	69.41	68.88	70.48	70.21	49.33	54.16	52.55	52.01	49.06	49.87	52.96	53.91	54.69	55.50	55.50
Pser4	53.64	56.95	54.50	56.13	56.40	56.68	53.26	54.35	55.98	55.98	64.53	61.01	60.21	60.74	89.66	100.00	94.96	94.16	72.61	72.07	72.87	72.61	50.94	54.42	52.28	51.74	47.98	50.94	52.15	52.83	54.42	55.23	55.23
Pser2	55.53	56.40	54.22	55.86	56.13	57.77	54.35	55.43	57.07	57.07	66.40	62.60	61.54	62.07	87.27	94.96	100.00	97.61	75.53	75.00	75.27	75.00	51.74	55.50	53.62	53.08	46.90	50.67	53.23	53.91	55.23	56.03	56.03
Pser1	54.72	56.40	54.22	55.59	56.13	57.77	54.35	55.43	57.07	57.07	66.67	62.86	61.80	62.33	87.00	94.16	97.61	100.00	75.00	74.47	75.00	74.73	51.74	54.42	52.55	52.55	47.17	49.60	52.96	54.18	54.96	55.76	55.76
Ppap1	51.89	53.55	50.55	52.19	52.46	55.19	51.23	53.95	54.50	54.50	60.16	57.45	57.18	57.71	69.41	72.61	75.53	75.00	100.00	99.20	84.04	83.78	48.92	50.00	51.88	50.54	44.05	48.25	50.67	48.38	49.73	50.27	50.27
Ppap3	51.35	53.55	50.55	52.19	52.46	55.46	51.50	54.22	54.77	54.77	59.36	57.18	56.65	57.18	68.88	72.07	75.00	74.47	99.20	100.00	83.51	83.24	48.39	49.46	51.88	50.81	44.32	47.98	50.40	48.11	49.46	50.00	50.00
Pdub3	51.89	52.46	50.55	51.64	53.01	55.74	52.04	53.13	54.22	54.22	59.63	57.18	56.12	56.65	70.48	72.87	75.27	75.00	84.04	83.51	100.00	98.41	49.19	51.34	51.61	51.08	45.95	49.06	50.13	50.00	50.81	51.61	51.61
Pdub2	52.16	52.46	50.55	51.91	53.01	55.19	51.77	52.86	54.22	53.95	59.89	57.98	56.65	57.18	70.21	72.61	75.00	74.73	83.78	83.24	98.41	100.00	49.46	51.61	51.08	50.81	45.41	48.52	50.40	50.00	50.81	51.61	51.61
Llon2	48.28	45.80	44.17	45.38	46.34	49.46	49.33	47.45	48.93	48.40	49.47	48.81	48.94	48.94	49.33	50.94	51.74	51.74	48.92	48.39	49.19	49.46	100.00	68.22	63.14	61.76	44.30	45.87	49.74	49.07	51.32	52.11	52.11
Laya4	55.97	53.66	51.49	53.26	55.01	59.14	56.57	56.84	56.95	56.42	53.17	51.99	53.17	53.44	54.16	54.42	55.50	54.42	50.00	49.46	51.34	51.61	68.22	100.00	74.68	72.61	47.75	50.67	53.70	52.25	54.74	55.79	55.79
Lint	54.11	53.12	51.76	52.45	53.39	58.60	54.69	54.42	56.68	56.15	50.79	49.60	52.12	52.12	52.55	52.28	53.62	52.55	51.88	51.88	51.61	51.08	63.14	74.68	100.00	79.33	47.75	52.80	51.59	49.87	53.42	54.47	54.47
Lolm3	53.85	53.39	50.95	52.45	52.85	59.68	56.03	56.84	58.29	55.61	51.85	49.87	51.32	51.32	52.01	51.74	53.08	52.55	50.54	50.81	51.08	50.81	61.76	72.61	79.33	100.00	49.60	49.87	52.12	53.58	54.47	55.53	55.53
Llon3	48.66	49.59	51.23	51.09	51.50	50.68	50.00	49.18	50.00	50.27	46.92	45.19	45.72	46.26	49.06	47.98	46.90	47.17	44.05	44.32	45.95	45.41	44.30	47.75	47.75	49.60	100.00	54.40	60.05	57.67	62.01	62.80	63.06
Lolm1	47.98	50.82	48.91	49.73	50.00	52.73	50.95	51.23	50.14	52.59	51.48	51.21	50.67	51.21	49.87	50.94	50.67	49.60	48.25	47.98	49.06	48.52	45.87	50.67	52.80	49.87	54.40	100.00	57.98	60.80	61.54	62.86	62.86
3Q6K_Llon1	53.62	52.45	51.36	52.86	51.09	54.62	51.76	52.30	52.85	52.30	49.87	49.60	49.60	50.13	52.96	52.15	53.23	52.96	50.67	50.40	50.13	50.40	49.74	53.70	51.59	52.12	60.05	57.98	100.00	70.90	78.42	79.47	79.74
Lolm2	50.27	49.86	49.05	50.55	49.32	53.13	51.63	51.36	52.17	51.36	51.47	51.87	51.34	51.34	53.91	52.83	53.91	54.18	48.38	48.11	50.00	50.00	49.07	52.25	49.87	53.58	57.67	60.80	70.90	100.00	78.89	79.42	79.68
Laya1	53.60	51.22	50.68	51.63	50.68	53.78	52.56	52.02	53.10	52.02	52.53	54.11	53.32	53.85	54.69	54.42	55.23	54.96	49.73	49.46	50.81	50.81	51.32	54.74	53.42	54.47	62.01	61.54	78.42	78.89	100.00	98.43	98.69
Laya2	54.13	52.03	51.49	52.45	51.49	54.32	53.10	52.56	53.64	52.56	53.60	55.17	54.38	54.91	55.50	55.23	56.03	55.76	50.27	50.00	51.61	51.61	52.11	55.79	54.47	55.53	62.80	62.86	79.47	79.42	98.43	100.00	99.74
Laya3	54.13	52.03	51.49	52.45	51.49	54.32	53.10	52.56	53.64	52.56	53.60	55.17	54.38	54.91	55.50	55.23	56.03	55.76	50.27	50.00	51.61	51.61	52.11	55.79	54.47	55.53	63.06	62.86	79.74	79.68	98.69	99.74	100.00

40.00-49.99%
50.00-59.99%
60.00-69.99%
70.00-79.99%
80.00-89.99%
90.00-99.99%
